# Differences in Zinc Content in Preterm Human Milk May Depend on Your Definition of “Preterm”

**DOI:** 10.1016/j.advnut.2025.100435

**Published:** 2025-05-22

**Authors:** Brian Stansfield, Amy Gates

**Affiliations:** Department of Pediatrics, Augusta University Medical College of Georgia, GA

Dear Editor:

We read with great interest the review published by Consales et al. [1] entitled “Tracing Zinc’s Role in Preterm Infants’ Health: A Narrative Review.” Zinc repletion for preterm and term infants is gaining significant attention, with several clinical trials demonstrating improved linear growth and less morbidity [2]. The review is timely and richly informative as a guide for clinicians who seek to provide sufficient zinc content to preterm and term infants.

The authors highlight an existing controversy regarding the expression of zinc in preterm human milk compared with term human milk. Zinc content in human milk is greatly influenced by lactation stage, with a rapid decline observed over the first 2 mo after birth. Given the relationship between zinc concentration and lactation stage, it is reasonable to observe conflicting results when other comparisons or covariates are considered. In line with this supposition, comparisons of zinc content between preterm and term human milk have demonstrated both higher and lower zinc content in preterm human milk, leading the authors of this review to agree with the conclusions of a recent systematic review [3] that “no significant differences in zinc concentrations between preterm and term human milk” are observed unless stage of lactation is considered as a covariate. Accounting for time since birth, the authors of the systematic review report that mean zinc concentration for preterm human milk is 3.37 mg/L, which is ∼25% higher than term human milk.

Unfortunately, Consales et al. did not include our manuscript that reports zinc concentration in human milk from 38 mothers who delivered preterm at a mean gestational age at birth of 28 wk [4]. We identified a predictable decrease in zinc concentration from 5 mg/L to 3 mg/L over the first 28 d after birth for this cohort. Although these values are within the reported reference range for zinc based on stage of lactation, we recently re-examined this data to account for gestational age as a potential explanatory variable for differences in zinc concentrations in preterm human milk. We did not identify a correlation between gestational age at birth (range 22–34 wk) and zinc concentration in human milk at the same timepoint after birth (days 7, 14, 21, and 28). Figure 1A demonstrates day of life 7 and 28 as examples. If we convert gestational age into a dichotomous variable at 28 wk postmenstrual age (i.e., <28 wk and ≥28 wk), we can identify modest differences in zinc concentrations between extremely low gestational age newborns (ELGANs, <28 wk) during the first 2 wk of postnatal life compared with older preterm infants (Figure 1B). These potential differences quickly merge and largely support the conclusions by Consales et al. [1] that lactation stage is the major driver of zinc content in human milk.

**FIGURE 1:**
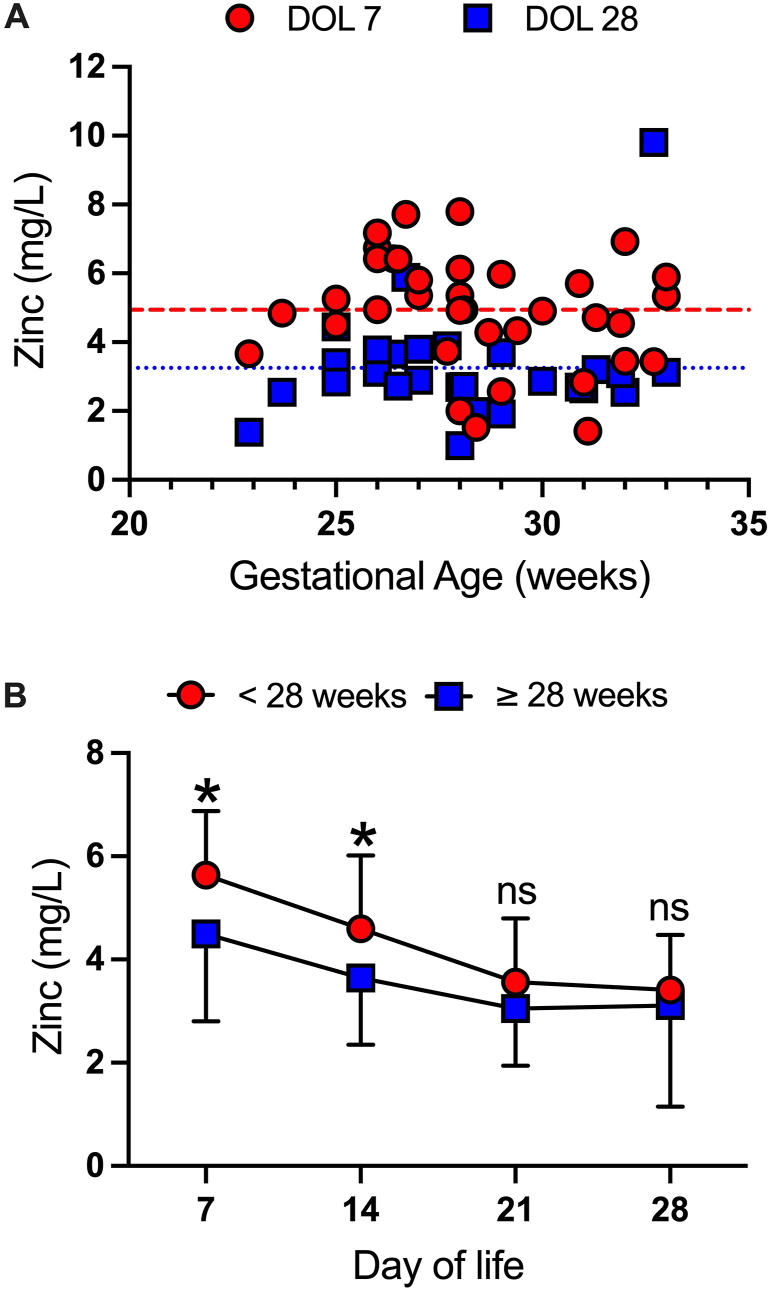
Zinc content in preterm human milk. (A) Zinc concentration (mg/L) at day of life (DOL) 7 (red circles) and 28 (blue squares) in preterm human milk across gestational age (wk) at birth. Red dashed line and blue dotted line represent mean zinc concentration at DOL 7 and 28, respectively. (B) Zinc concentration (mg/L) at DOL 7, 14, 21, and 28 in preterm human milk by dichotomous gestational age (wk) at birth. Preterm infants born < 28 wk (red circles) are compared to preterm infants born ≥ 28 wk (blue squares). Data represents mean ± SD. ∗*P* < 0.05, *NS* = not significant. Analysis by Student’s *t* test.

Our results indicate potential differences in zinc content based on the use of a dichotomous variable that are not observed with continuous variables. Preterm infants are often categorized using gestational age cutoffs such as <28 wk, which may partly explain the heterogeneity of results related to zinc as well as other macro and micronutrients.

## Funding

Data was generated from an unrestricted grant from Mead Johnson Nutrition.

## Conflict of interest

BS reports a relationship with Mead Johnson Nutrition that includes grant funding and membership in the speaker’s bureau. AG works for Mead Johnson Nutrition.
